# Ruxolitinib Improves Immune-Dysregulation Features but not Epigenetic Abnormality in a Patient with STAT1 GOF

**DOI:** 10.1007/s10875-024-01687-9

**Published:** 2024-04-05

**Authors:** June-Young Koh, Doo Ri Kim, Sohee Son, Hwanhee Park, Kyung-Ran Kim, Sunwoo Min, Ha Seok Lee, Byung Woo Jhun, Eun-Suk Kang, Inkyung Jung, Ji-Man Kang, Yae-Jean Kim, Eui-Cheol Shin

**Affiliations:** 1 Graduate School of Medical Science and Engineering, Korea Advanced Institute of Science and Technology (KAIST), Daejeon, 34141 Republic of Korea; 2 Genome Insight, Inc., Daejeon, 34051 Republic of Korea; 3 Department of Pediatrics, Samsung Medical Center, Sungkyunkwan University, Seoul, 06351 Republic of Korea; 4 Department of Pediatrics, Soonchunhyang University Bucheon Hospital, Soonchunhyang University College of Medicine, Bucheon, 14584 Republic of Korea; 5 Department of Pediatrics, Gyeongsang National University Changwon Hospital, Gyeongsang National University College of Medicine, Changwon, 51472 Republic of Korea; 6 Department of Biological Sciences, Korea Advanced Institute of Science and Technology (KAIST), Daejeon, 34141 Republic of Korea; 7 Division of Pulmonary and Critical Care Medicine, Department of Medicine, Samsung Medical Center, Sungkyunkwan University School of Medicine, Seoul, 06351 Republic of Korea; 8 Department of Laboratory Medicine and Genetics, Samsung Medical Center, Sungkyunkwan University School of Medicine, Seoul, 06351 Republic of Korea; 9 Department of Pediatrics, Severance Children’s Hospital, Yonsei University College of Medicine, Seoul, 03722 Republic of Korea; 10 Institute for Immunology and Immunological Diseases, Yonsei University College of Medicine, Seoul, 03722 Republic of Korea; 11 The Center for Viral Immunology, Korea Virus Research Institute, Institute for Basic Science (IBS), Daejeon, 34126 Republic of Korea

**Keywords:** Inborn errors of immunity, STAT1 GOF, JAK inhibitor, ATAC sequencing

## Abstract

**Purpose:**

Patients with STAT1 gain-of-function (GOF) mutations often exhibit autoimmune features. The JAK1/2 inhibitor ruxolitinib can be administered to alleviate autoimmune symptoms; however, it is unclear how immune cells are molecularly changed by ruxolitinib treatment. Then, we aimed to investigate the trnscriptional and epigenetic status of immune cells before and after ruxolitinib treatment in a patient with STAT1 GOF.

**Methods:**

A patient with a heterozygous STAT1 GOF variant (p.Ala267Val), exhibiting autoimmune features, was treated with ruxolitinib, and peripheral blood mononuclear cells (PBMCs) were longitudinally collected. PBMCs were transcriptionally analyzed by single-cell cellular indexing of the transcriptomes and epitopes by sequencing (CITE-seq), and epigenetically analyzed by assay of transposase-accessible chromatin sequencing (ATAC-seq).

**Results:**

CITE-seq analysis revealed that before treatment, the patient’s PBMCs exhibited aberrantly activated inflammatory features, especially IFN-related features. In particular, monocytes showed high expression levels of a subset of IFN-stimulated genes (ISGs). Ruxolitinib treatment substantially downregulated aberrantly overexpressed ISGs, and improved autoimmune features. However, epigenetic analysis demonstrated that genetic regions of ISGs—e.g., *STAT1*, *IRF1*, *MX1*, and *OAS1*—were highly accessible even after ruxolitinib treatment. When ruxolitinib was temporarily discontinued, the patient’s autoimmune features were aggravated, which is in line with sustained epigenetic abnormality.

**Conclusions:**

In a patient with STAT1 GOF, ruxolitinib treatment improved autoimmune features and downregulated aberrantly overexpressed ISGs, but did not correct epigenetic abnormality of ISGs.

**Supplementary Information:**

The online version contains supplementary material available at 10.1007/s10875-024-01687-9.

## Introduction

Janus kinase (JAK)-signal transducer of activators of transcription (STAT) pathways play crucial roles in the intracellular signal transduction of various cytokines, including interferons (IFNs) [1–4]. Genetic mutations in JAK-STAT-related molecules can lead to uncontrolled activation of downstream genes, resulting in clinical abnormality.

STAT1 gain-of-function (GOF) mutations cause an autosomal-dominant syndrome with variable clinical features [5, 6]. The most common symptom is early-onset chronic mucocutaneous candidiasis (CMC), but a spectrum of various autoimmune and autoinflammatory features have also been reported [7, 8]. In recent years, JAK inhibitors (JAKi) have been therapeutically used to treat patients with STAT1 GOF [9–12]. Although JAKi treatment has yielded clinical benefits in several cases, it remains unclear what molecular events are elicited by JAKi.

Here, we longitudinally examined transcriptional and epigenetic changes in immune cells of a patient with STAT1 GOF, over a course of ruxolitinib treatment. To this end, we performed single-cell multi-omics analyses, including simultaneous profiling of proteins and RNA with cellular indexing of the transcriptomes and epitopes by sequencing (CITE-seq), and assay of transposase-accessible chromatin sequencing (ATAC-seq).

## Methods

### Subject Detail

This research was reviewed and approved by the institutional review board of Samsung Medical Center (IRB No. 2018–07-140). Informed consent was obtained from the study participant.

### Cell Preparation

Peripheral blood mononuclear cells (PBMCs) were separated by density gradient centrifugation using lymphocyte separation medium (Corning), and cryopreserved in fetal bovine serum (FBS; Corning) with 10% DMSO (Sigma-Aldrich).

### ELISA

Serum from the patient and healthy donors assessed for CXCL10 concentration using the human Recombinant Human CXCL10/IP-10 Protein ELISA kit (R&D) according to the manufacturer’s instructions.

### FACS Analysis

Cryopreserved PBMCs were thawed and subjected to staining with fluorochrome-conjugated antibodies against surface markers for 10 min at room temperature. Dead cells were excluded from analysis using LIVE/DEAD red/aqua fluorescent reactive dye (Invitrogen, Waltham, MA, USA). Multicolor flow cytometry was carried out using an LSR II instrument (BD Biosciences, Franklin Lakes, NJ, USA), and the data were analyzed using FlowJo software (BD Biosciences).

### Methanol Fixation

PBMCs were labeled with antibodies targeting surface markers. Subsequently, cells were exposed to IFN-β (50 ng/mL) or PBS for 10 min at 37 °C. This was followed by treatment with IC fixation buffer (Invitrogen) for 10 min at room temperature and fixation using 100% cold methanol for 40 min at 4 °C. Afterward, cells were stained with anti-human STAT1 antibodies for 1 h at room temperature before undergoing flow cytometry analysis.

### Single-Cell RNA-seq Library Preparation

Single-cell CITE-seq and ATAC-seq libraries were generated using the Chromium Next GEM Automated Single Cell 3′ Library and Gel Bead Kit v3.1 (PN-1000147), 3ʹ Feature Barcode Kit (PN-1000262) and Chromium Next GEM Single Cell ATAC Kit v2 (PN-1000406), following the manufacturer’s instructions. Libraries were constructed and sequenced at a depth of approximately 20,000 reads per cell for gene, antibody-derived tag (ADT), and ATAC-seq fragment. The sequenced data were demultiplexed using the mkfastq function (Cell Ranger, 10X Genomics, v3.0.1) to generate fastq files. Demultiplexed fastq files of gene and ADT expression were aligned to the reference human genome (GRCh38; 10X Cell Ranger reference GRCh38 v3.0.0) and the barcode sequence of each ADT. Filtering, barcode counting, and UMI counting were performed to generate feature-barcode matrices using the count function (Cell Ranger, v3.0.1).

### Single-Cell RNA-seq Analysis

The feature-barcode matrices of RNA and ADT expression were analyzed as follows, using the Seurat R package (Seurat, v4.1.1) [13]. For basic quality control, we de-convoluted sample identity and filtered inter-individual multiplets using the demuxlet package [14]. Next, we filtered low-quality cells expressing mitochondrial genes in > 15% of their total gene expression, or in > 5,000 genes. We also excluded doublets that initially clustered with doublets annotated through the demuxlet package.

Subsequently, the sctransform method (SCTransform function from sctransform package, v0.3.3) [15] was used to perform normalization and variance stabilization of the RNA read count matrix with highly variable genes (*n* = 2,000), and of the feature-barcode read count matrix with the whole list of feature barcodes. Principal component analysis (PCA; RunPCA function) was carried out to dimensional reduction for each transformed data matrix. Next, the cells underwent unsupervised clustering according to the weighted nearest neighbor (WNN) graph—applying the FindMultiModalNeighbors function, using the top 20 principal components (PCs) from RNA, and 20 PCs from feature barcode, along with the FindClusters function (resolution = 0.4). The results were visualized by Uniform Manifold Approximation and Projection (UMAP) using the top 20 PCs from RNA for whole PBMCs. For sub-clustering analysis of the monocyte immune subset, the cells underwent unbiased clustering, performed using the top 25 PCs from RNA and 15 PCs (resolution = 0.3) from feature barcode, and the results were visualized by UMAP using the top 20 PCs from RNA. To identify marker genes, differentially expressed genes (DEGs) in each cluster relative to the other clusters were selected based on the Wilcoxon rank sum test, using the FindAllMarkers function (parameter; log fold change compared to the other clusters > 0.25, > 0.6 min.pct2 [minimum fraction of test genes detected in cells of other clusters], and Bonferroni-adjusted *P* < 0.05).

To describe the characteristic of each subcluster, we performed gene set enrichment analysis by calculating the gene set module score (AddModuleScore in Seurat package), combined score (enrichR), [16] and enrichment score (GSEA v4.2.3, Broad Institute, CA, USA) using publicly available gene sets, including Gene Ontology: Biological process databases (GO.BP) [17] and LINCS L1000 data [18]. We filtered Gene Ontology (GO): Biological Process (BP) terms related to immune responses using the following inclusion criteria: "T_CELL", "IMMUNE", "INNATE", "ADAPTIVE", "INFLAM", "INTERLEUKIN", "INTERFERON", "NECROSIS", "APOPTOSIS", "SENESCENCE", "NATURAL", "LYMPHOCYTE", "LEUKOCYTE", "TRANSFORMING", "CHEMO", "CYTOTO", "CYTOKINE", "ANTIGEN", and "SIGNALING" and TRRUST TF. To investigate the dynamic changes of the monocyte immune subset, we exported cells from the monocyte immune subset for monocle’s standard analysis process (monocle v2.22.0) [19]. CellDataSet objects were built based on the normalized count (SCTransform), and then processed using the estimateSizeFactor and estimate-Dispersions functions (default option), detectGenes (default option), setOrdering-Filter and reduceDimension (default option), orderCells (default option), and plot_cell_tra-jectory (default option).

### Single-Cell ATAC-seq Analysis

The peak-cell matrices were analyzed as follows, using the Signac R package (Signac, v1.8.0) [20]. For basic quality control, we calculated the nucleosome binding pattern, transcriptional start site (TSS) enrichment score that represents the accessibility signal over all TSS sites, and the ratio of reads in genomic blacklist regions. Next, we filtered low-quality cells using the ratio of mononucleosomal to nucleosome-free fragments (> 3), TSS enrichment (< 2), and ratio of reads in genomic blacklist regions (> 0.05). On the filtered matrix, we performed normalization and linear dimensional reduction using term frequency-inverse document frequency (TF-IDF) normalization, followed by singular value decomposition (SVD) using the top 25% of all peaks. Through this process, we calculated the latent semantic indexing (LSI) component (RunTFIDF, FindTopFeatures, and RunSVD function) [21]. To integrate and correct the batch effect across each sample, we found integration anchors and integrated the datasets using reciprocal LSI projection. We calculated the gene activity score, which predicts and quantifies the activity of each gene in the genome, by assessing the chromatin accessibility associated with each gene, using the GeneActivity function.

### Chip-qPCR

Collected PBMCs from healthy donors and a patient with STAT1 GOF mutation were cross-linked with 1% formaldehyde for 10 min and quenched with 125 mM glycine for 5 min at room temperature. The cross-linked PBMCs were lysed with 1% SDS buffer (1% SDS, 10 mM EDTA, 50 mM Tris pH 8.1) and chromatin was fractionated by sonication (Covaris, S220). The lysate was diluted with dilution buffer and incubated with pre-coated Dynabeads Protein A (Thermo, 10001D) with H3K27ac antibody (Active Motif, 39,133) and IgG as negative control overnight at 4ºC with rotation. A portion of diluted lysate was stored to quantify the total amount of DNA in each sample as input control. The immunoprecipitated chromatin was washed four times with RIPA buffer (140 mM NaCl, 1 mM EDTA, 0.5 Mm EGTA, 1% Triton X-100, 0.1% SDS, 0.1% sodium deoxycholate, 10 mM Tris pH 8.0) and incubated with RNase A (Thermo, EN0531) and proteinase K (NEB, P8107S) overnight at 65 ºC to reverse crosslink. The immunoprecipitated DNA was purified by AMPure XP beads (Beckman, A63881) and analyzed by Real-Time PCR using ChIP-qPCR primers (STAT1: FW – ACTCTGCGCAGGAAAGCGAA, RV – GGAACAGCCGCGTCTAATTG; IRF1: FW- AGCCAAGGAATTGCTCCAGT, RV – ACAGGGGGCTGAGTTCTCTT).

### Resource Availability

Further information and requests for resources and reagents should be directed to and will be fulfilled by the lead contact, Eui-Cheol Shin (ecshin@kaist.ac.kr).

### Data and Code Availability

CITE-seq data and ATAC-seq will be deposited at the NCBI GEO (https://www.ncbi.nlm.nih.gov/geo/). This study did not generate any original code. Links to the publicly available R packages and codes used in the paper are listed in the key resources table. Any additional information required to reanalyze the data reported in this paper is available from the lead contact upon request.

## Results

### Autoimmune Features in a Patient with STAT1 GOF and Ruxolitinib Treatment

**Fig. 1:**
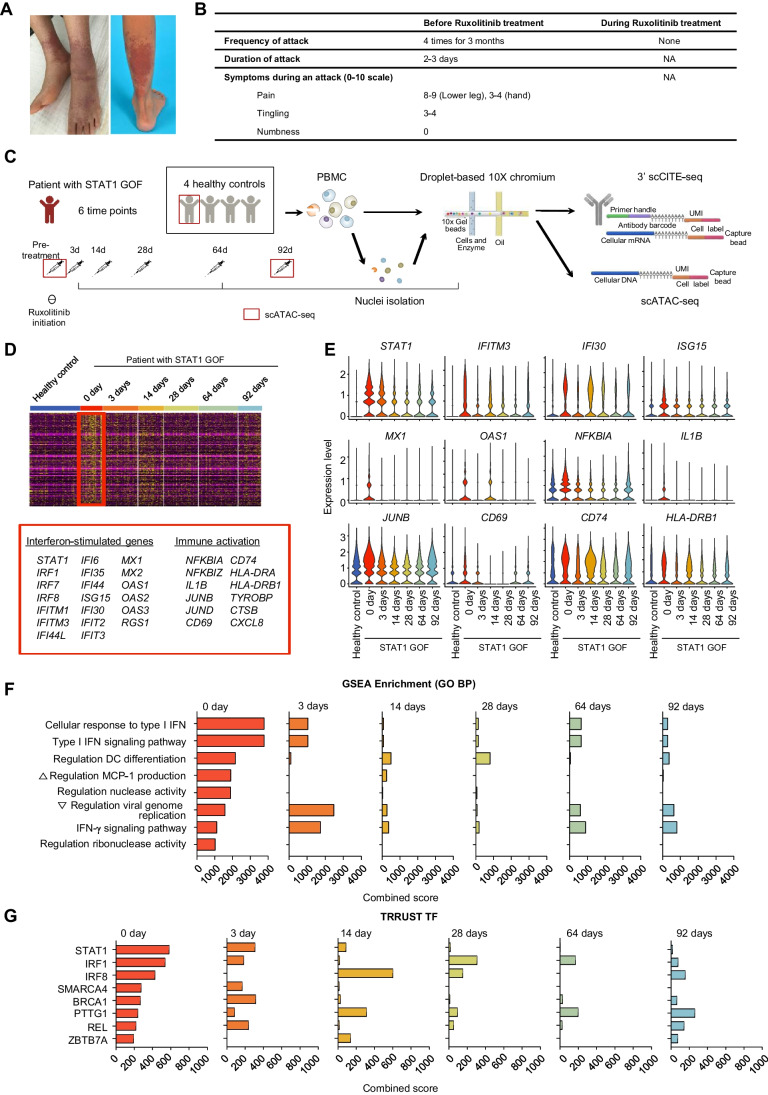
Longitudinal multi-omics analysis of peripheral blood mononuclear cells (PBMCs) from a patient with STAT1 gain-of-function (GOF) mutation, before and after ruxolitinib treatment. **A** Medical photos showing Raynaud’s phenomenon in her feet. **B** Disease activity and functional status of patient’s Raynaud’s phenomenon before ruxolitinib treatment. **C** Summary of the experimental design. **D** Heatmap showing differentially expressed genes (DEGs) of PBMCs from the patient with STAT1 GOF before ruxolitinib treatment, compared to healthy controls. **E** Violin plots showing expressions of representative genes at each time-point in the patient and in healthy controls. **F-G** Bar plots showing gene set enrichment analysis (GSEA) score of GO BP (**F**) and TRRUST TF (**G**) at each time-point in the patient and in healthy controls. NA, not applicable

We previously reported the case of a 23-year-old woman with a heterozygous STAT1 GOF variant (NM_007315.3:c.800C > T, p.Ala267Val), which was a known pathogenic variant, who presented with bronchiectasis and recurrent respiratory infections (Supplementary Fig. 1A and B) [22]. The variant has previously been reported to cause both delayed dephosphorylation of phosphorylated STAT1 (pSTAT1) [23] and increased STAT1 expression levels [24, 25]. We confirmed that the STAT1 expression levels were increased in PBMCs or monocytes from the patient compared to in healthy donors (Supplementary Fig. 1C). She exhibited autoimmune symptoms, with severe Raynaud’s phenomenon in her hands and feet and tenosynovitis in multiple joints (Fig. 1A and B) [26]. Her rheumatoid factor level was elevated to 84.3 IU/mL (reference range, < 14 IU/mL), and she was weakly positive for fluorescent antinuclear antibody intermittently. Hydroxychloroquine treatment was administered for 22 months, but her autoimmune symptoms persisted. We next initiated treatment with the oral JAK1/2 inhibitor ruxolitinib (10 mg per dose twice a day; body weight, 33 kg), which yielded dramatic disappearance of the autoimmune symptoms. Notably, Raynaud’s phenomenon did not develop for seven months after starting ruxolitinib treatment.

### Regulation of Aberrant Immune Responses at the Transcriptional Level by Ruxolitinib Treatment

Peripheral blood mononuclear cells (PBMCs) were longitudinally collected from the patient before and after ruxolitinib treatment. PBMCs were also obtained from four healthy donors. We used the Chromium system (10 × Genomics) to perform CITE-seq, which allows simultaneous profiling of protein markers and RNA, and ATAC-seq (Fig. 1C). First, we performed pseudo-bulk analysis, using CITE-seq data to identify transcriptional features of whole PBMCs from the patient with STAT1 GOF before and after ruxolitinib treatment. We identified the differentially expressed genes (DEGs) in the patient’s PBMCs before treatment (day 0) compared to the healthy controls’ PBMCs (Fig. 1D and Supplementary Fig. 2A-C). The pre-treatment DEGs featured IFN-stimulated genes (ISGs) (e.g., *STAT1*, *ISG15*, *MX1,* and *OAS1*) and immune activation genes (e.g., *NFKBIA* and *JUNB*), and the expression levels of these genes substantially decreased after ruxolitinib treatment (Fig. 1E). Next, we performed gene set enrichment analysis (GSEA) using the Gene Ontology Biological Process and TRRUST TF databases. The pre-treatment DEGs were significantly associated with type I and type II IFN pathway-related gene sets and transcription factors (e.g., STAT1 and IRF1), and the combined scores of these gene sets decreased after ruxolitinib treatment as early as 3 days (Fig. 1F and G). We also calculated differentially expressed proteins (DEPs) in our patient at day 0 compared to in healthy donors, to identify cell surface proteins that were upregulated in the patient with STAT1 GOF. At day 0, we found significant upregulation of chemokine receptors, such as CX3CR1 and CD183 (CXCR3), and co-stimulatory molecules, such as CD80 and CD86. These proteins were downregulated in the patient after JAKi treatment. (Supplementary Fig. 3A and B). We also performed ELISA to determine the serum levels of CXCL10, a representative chemokine that is inducible by type I or II IFNs. Compared to healthy donors, our patient with STAT1 GOF exhibited a marked increase of *CXCL10* transcript and CXCL10 serum levels, which gradually decreased after JAKi treatment. (Supplementary Fig. 3C and D). Collectively, the PBMCs from a patient with STAT1 GOF exhibited aberrantly activated inflammatory features, especially IFN-related features, and these aberrant features were effectively down-modulated by ruxolitinib treatment.

### Downregulation of IFN-Responsive Genes in Monocytes by Ruxolitinib Treatment

**Fig. 2:**
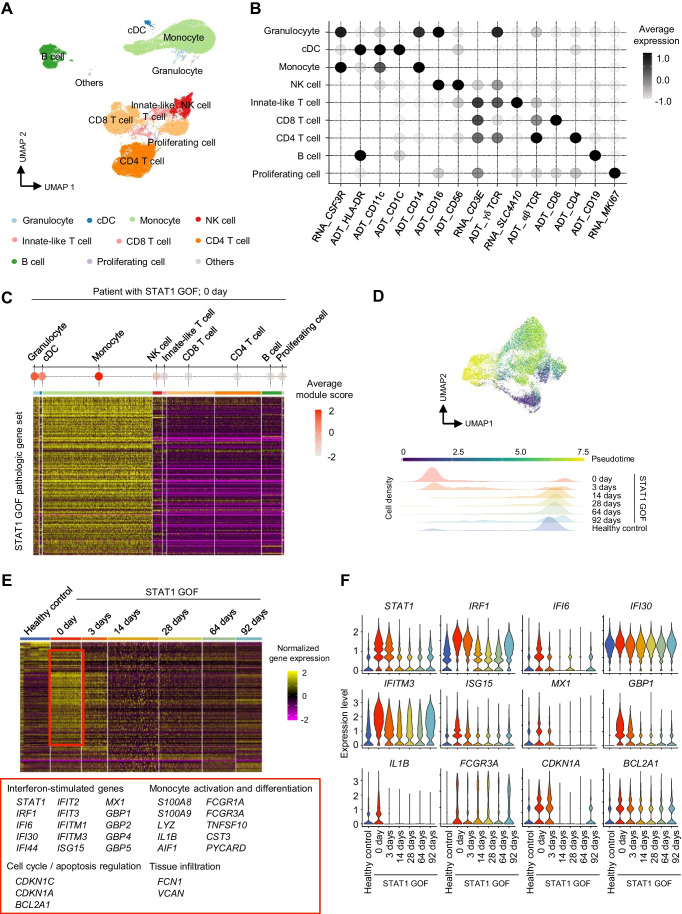
Dynamic transcriptomic changes of the monocyte population of a patient with STAT1 gain-of-function (GOF) mutation. **A** Uniform Manifold Approximation and Projection (UMAP) of peripheral blood mononuclear cells (PBMCs) from healthy controls (*n* = 4) and a patient with STAT1 GOF, at 6 time-points. **B** Dot plots showing the average expression of marker genes and proteins in each immune cell cluster. **C** Dot plot and heatmap showing average gene set module scores and normalized gene expression in each immune cell cluster from the patient with STAT1 GOF before ruxolitinib treatment. **D** Plots showing the cell density of each sample across pseudotime. **E** Heatmap showing differentially expressed genes (DEGs) in monocytes, calculated at each time-point, in the patient with STAT1 GOF compared to healthy controls. **F**, Violin plots showing expression of representative genes in monocytes at each time-point in the patient and in healthy controls

Next, we performed cell clustering, and analyzed each immune cell cluster using the WNN method, which enables the simultaneous consideration of both surface protein expression and transcriptional expression [27]. According to the expressions of genes and markers stained by antibody-derived tags (ADTs), we identified the following nine immune cell types (Fig. 2A): granulocytes, cDCs, monocytes, NK cells, innate-like T cells, CD8 T cells, CD4 T cells, B cells, and proliferating cells (Fig. 2B). We examined which immune cell types mainly exhibited aberrantly activated inflammatory features before treatment (day 0). To this end, we calculated the gene set module scores of each immune cell type, using the DEGs of PBMCs from the pre-treatment time-point, and found that the monocyte population showed the highest enrichment of the DEGs from pre-treatment PBMCs (Fig. 2C).

We subsequently focused on the dynamic transcriptomic changes of the monocyte population after ruxolitinib treatment. In pseudotime analysis, we identified a pseudotime trajectory with monocytes from pre-treatment and day 3 aligned at an earlier pseudotime, and monocytes from healthy controls and the other post-treatment groups (days 14, 28, 64, and 92) aligned at a later pseudotime (Fig. 2D). These findings validated ruxolitinib-induced transcriptomic changes among monocytes. The DEGs of pre-treatment monocytes featured genes related to IFN response (*STAT1*, *IRF1,* and *ISG15*), monocyte activation and differentiation (*S100A8, S100A9*, *IL1B,* and *FCGR3A*), cell cycle and apoptosis regulation (*CDKN1C* and *BCL2A1*), and tissue infiltration (*FCN1* and *VCAN*). The expressions of these genes substantially decreased after ruxolitinib treatment (Fig. 2E and F).

### Modulation of a Subset of ISGs

**Fig. 3:**
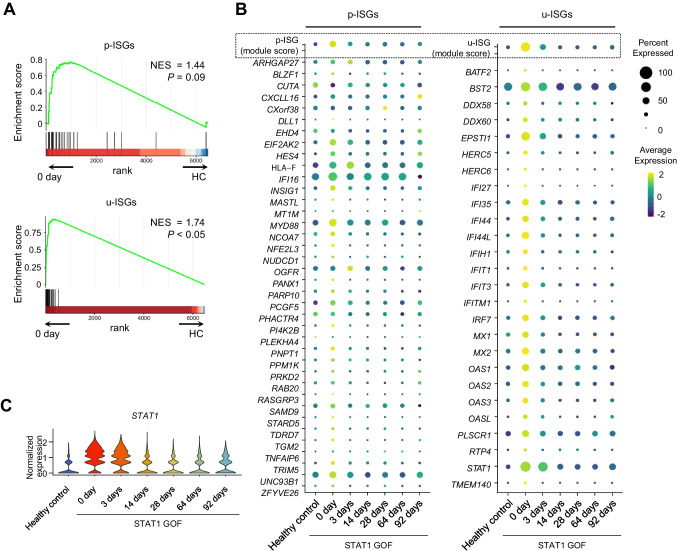
Expressions of two different sets of interferon (IFN)-stimulated genes in the monocyte population of a patient with STAT1 gain-of-function (GOF) mutation. **A** Gene set enrichment analysis (GSEA) of genes that were up-regulated in the monocyte population of the patient with STAT1 GOF at day 0 (before treatment) compared to in healthy controls, stratified by the gene sets related to p-ISGs versus u-ISGs. **B** Dot plots showing average gene set module scores and gene expression of u-ISGs in monocytes, from each time-point, in the patient and in healthy controls. **C** Violin plots showing *STAT1* expression of monocytes from each time-point from the patient and the healthy controls

We further analyzed the expressions of two different sets of ISGs. Type I IFN stimulation deliver signals via phosphorylated ISGF3 (p-ISGF3) formed by p-STAT1, p-STAT2, and IRF9 [28]. On the other hand, prolonged type I IFN stimulation leads to STAT1 upregulation, which can form ISGF3 even without phosphorylation, so-called unphosphorylated ISGF3 (u-ISGF3) [29, 30]. Downstream genes of p-ISGF3 and u-ISGF3 are known as p-ISGs (ISGs uniquely induced by p-ISGF3) and u-ISGs (ISGs commonly induced by p-ISGF3 and u-ISGF3), respectively [29–31]. Based on this background, we examined which IFN pathway was more associated with aberrantly activated IFN-related features. In pre-treatment monocytes, we detected significantly enrichment of a gene set of u-ISGs (Fig. 3A). In contrast, after ruxolitinib treatment, a gene set of u-ISGs was substantially down-regulated. Notably, *STAT1* itself is part of a gene set of u-ISGs, and *STAT1* expression showed the same pattern observed for u-ISG expression (Fig. 3B and C). Next, we evaluated the expression levels of u-ISGF3 components in pre-treatment monocytes. We first examined the transcript levels of STAT1, STAT2, and IRF9, and found that monocytes from the patient exhibited upregulation of all three on day 0 (before JAKi treatment) compared to healthy donors (Supplementary Fig. 4A). We also examined the STAT1 protein levels using flow cytometry analysis. These results confirmed that the STAT1 protein levels were much higher in monocytes from the patient on day 0, compared to in healthy donors. Upon ex vivo IFN-β treatment, the STAT1 protein levels were greatly increased at 3 h, and then returned to the baseline level at 12 h, which was still higher than the levels in healthy donors (Supplementary Fig. 4B and C). These data indicated that STAT1 GOF led to persistent STAT1 upregulation, resulting in aberrant IFN features and that ruxolitinib treatment could successfully decrese the aberrant IFN-features.

### Persistence of Epigenetic Abnormality after Ruxolitinib Treatment

**Fig. 4:**
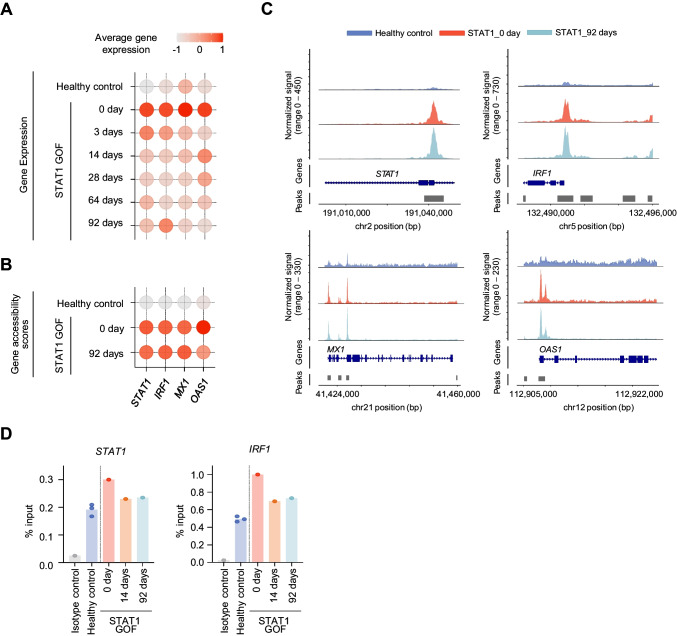
Retained epigenetic inflammatory features of immune cells from a patient with STAT1 gain-of-function (GOF) mutation after ruxolitinib treatment. **A-B** Dot plots showing gene expressions and gene accessibility scores of *STAT1, IRF1, MX1,* and *OAS1* from RNA-sequencing (**A**) and ATAC-sequencing (**B**), at each time-point, for the patient and the healthy controls. **C** Coverage plots showing each region’s genomic accessibility from each sample. **D** Bar graph showing the enrichment of H3K27ac at highly open chromatin regions near *STAT1* (left) and *IRF1* (right) in PBMC from patients with STAT1 GOF mutation before (day 0) and after treatment of JAK inhibitors (day 14 and day 92), compared to the level of H3K27ac in IFN-β stimulated PBMC from healthy donors as positive control. Enrichment of H3K27ac in y-axis was represented as the percentage of immunoprecipitated DNA with H3K27ac and IgG relative to input DNA analyzed by ChIP-qPCR using the indicated primers in methods section

Finally, we used ATAC-seq data to examine gene accessibility in before (day 0) and after (day 92) ruxolitinib treatment. Gene accessibility analysis provides important information regarding cellular differentiation and transition, because certain genes are not transcribed despite being highly accessible. In Fig. 4A, we present the gene expression levels of representative u-ISGs (including *STAT1*, *IRF1*, *MX1*, and *OAS1*) from PBMCs obtained during the course of treatment. The expression levels of *STAT1*, *IRF1*, *MX1*, and *OAS1* were dramatically reduced by ruxolitinib treatment (Fig. 4A); however, the gene accessibility scores of these genes were not decreased even at 92 days after starting ruxolitinib treatment (Fig. 4B). Coverage plots also demonstrated highly open chromatin regions near *STAT1*, *IRF1*, *MX1*, and *OAS1* in PBMCs of the patient with STAT1 GOF, both before (day 0) and after (day 92) ruxolitinib treatment (Fig. 4C). Furthermore, we used chromatin immunoprecipitation-qPCR (Chip-qPCR) to assess the differential occupancy of H3K27ac in target sites. H3K27ac is an epigenetic modification of histone H3, which is associated with enhanced transcription activation, and is thus defined as an active enhancer. Compared to healthy donors, PBMCs from the patient with STAT1 GOF at day 0 exhibited a higher signal intensity of H3K27ac in both the STAT1 and IRF1 regions, which was decreased after JAKi treatment (Fig. 4D). Overall, these results indicated that genetic regions of ISGs were highly accessible both before and after ruxolitinib treatment, although their transcriptional levels were no longer upregulated after ruxolitinib treatment. This suggested that discontinuation of ruxolitinib may enable the gene expression of ISGs to increase again, and aggravate autoimmune features.

Seven months after starting ruxolitinib treatment, the patient developed rapidly progressing pneumonia. Considering the possibility of pulmonary invasive fungal infection, liposomal amphotericin B was started and switched to posaconazole later. During this event, ruxolitinib was temporarily discontinued to avoid drug-drug interactions. One month after antifungal treatment initiation and ruxolitinib discontinuation, pneumonia resolved; however, the patient redeveloped Raynaud’s phenomenon in both ankles. When ruxolitinib treatment resumed, Raynaud’s phenomenon disappeared. The aggravation of autoimmune features following ruxolitinib discontinuation may be explained by the persistence of epigenetic abnormality, e.g., sustained high accessibility of ISG regions.

## Discussion

GOF mutations in the STAT1 gene result in an autosomal-dominant syndrome with diverse clinical manifestations. Patients with STAT1 GOF most commonly exhibit early-onset CMC and other bacterial, viral, and fungal infections, and may also experience autoimmune or autoinflammatory conditions. A large cohort study (*n* = 60) revealed autoimmune manifestations in over one-third of patients with STAT1 GOF [8]. These autoimmune manifestations can occur with varying degrees of severity, ranging from mild to life-threatening, and can affect diverse organs of the body. Potential conditions include hyperthyroidism, cutaneous diseases, systemic lupus erythematosus, inflammatory bowel disease, and IPEX-like symptoms [8, 32].

The management of patients with STAT1 GOF can be challenging due to the complex and varied nature of the manifestations—often requiring a balanced approach that utilizes both antimicrobial and immunosuppressive therapies. Even with such interventions, many patients fail to adequately respond and require multiple courses of immunosuppressive treatments [33]. In recent years, JAKi, small molecules that interfere with cytokine-dependent JAK-STAT pathways, have emerged as a promising treatment option for modulating aberrant immune responses in patients with STAT1 GOF [9–12]. Although JAKi treatment has yielded clinical benefits in several cases, the detailed mechanisms underlying the effects of JAKi treatment have not yet been fully elucidated.

In the current study, we performed single-cell multi-omics analysis (including CITE-seq and ATAC-seq) to describe the longitudinal changes of the immune landscape in a patient with STAT1 GOF receiving JAKi treatment (ruxolitinib). We found that the patient with STAT1 GOF exhibited aberrant overexpression of ISGs, particularly u-ISG, prior to treatment. Ruxolitinib treatment resulted in decreased expression of these aberrantly overexpressed ISGs, accompanied by symptom relief. However, despite this improvement, epigenetic abnormalities of ISGs persisted even after ruxolitinib treatment. Moreover, the autoimmune symptoms recurred after discontinuation of ruxolitinib treatment. These observations suggest that the persistence of epigenetic abnormalities (so-called epigenetic scars) could lead to a propensity for aberrant IFN responses, and may lower the threshold of inflammatory stimulation, resulting in prolonged autoimmune symptoms.

The signaling pathway for type I IFNs typically involves STAT phosphorylation, leading to the formation of p-ISGF3 (a trimer of p-STAT1, p-STAT2, and IRF9), which induces the expressions of various ISGs [34]. However, there is also evidence of non-canonical signaling that occurs through increased expression levels of STAT1, STAT2, and IRF9, rather than STAT phosphorylation [29, 35, 36]. This non-canonical signaling is observed in situations such as prolonged IFN stimulation, and yields the induction of u-ISGF3, which specifically activates a subset of ISGs, known as u-ISGs. u-ISGF3 is known to be formed by prolonged exposure to type I IFNs rather than type II IFNs [29, 30]. Interestingly, in our current study, the identified pretreatment DEGs displayed predominant expression of u-ISGs. Moreover, JAKi treatment resulted in significantly reduced expression levels of u-ISGs in both the monocyte subset and whole PBMCs. These results suggest that the defective STAT1 dephosphorylation observed in the patient with STAT1 GOF may result in prolonged and elevated expression of STAT1, which might in turn activate aberrant IFN signaling through the u-ISGF3 pathway. Ruxolitinib treatment was able to effectively regulate and terminate the aberrantly activated IFN signals in this patient.

Binding of type I and II IFNs to their receptors leads to the phosphorylation of JAKs, which then causes the recruitment and phosphorylation of downstream molecules, including STAT1 [3]. In patients with STAT1 GOF, higher pSTAT1 levels are observed after exposure to IFNs, which is a critical molecular feature of the disease [23]. Furthermore, increased pSTAT1 leads to upregulation of STAT1 itself. JAKi can suppress JAK phosphorylation, which is the cue initiating STAT1 phosphorylation and subsequent STAT1 upregulation; therefore, JAKi has recently been used for treatment of patients with STAT1 GOF [10, 12].

Our current results revealed that ruxolitinib treatment could not reverse the epigenetic features of activated IFN signals in the patient with STAT1 GOF. Technical advancements in next-generation sequencing and single-cell analysis enable examination of the transcriptomic features of heterogeneous cell populations under healthy and pathological conditions [37]. Disease-specific transcriptomic features may be resolved upon treatment. On the other hand, epigenetic changes are usually sustained even after clinical and transcriptional resolution. In a study using chromatin immunoprecipitation (ChIP) analysis, Epp et al. recently reported that PBCMs from patients with STAT1 GOF were significantly enriched with active chromatin mark trimethylation of lysine 4 of histone 3 (H3K4me3) in areas associated with ISGs, and that these epigenetic ISG signatures were retained even under low levels of serum IFN-α [38]. Our current results showed that even after clinical resolution of autoimmune features following JAKi treatment, aberrantly activated epigenetic ISG signatures were retained, along with increased H3K27ac signal intensity in regions of ISGs, such as *STAT1* and *IRF1*. These findings suggest the value of investigating treatment with agents, such as HDAC activators, that can restrict the prolonged increase of chromatin accessibility, and thereby improve clinical outcomes.

Recently, JAKi have been used to regulate pathological inflammatory responses in patients with various autoimmune diseases, including STAT1 GOF. However, there are not yet firmly established strategies for drug maintenance and dosage adjustment. Herein, we described the clinical and transcriptomic resolution of autoimmune features following ruxolitinib treatment in a patient with STAT1 GOF. Our ATAC-seq results revealed that this patient’s immune cells retained epigenetic scars even after ruxolitinib treatment. These findings help us understand the molecular mechanisms underlying the effects of JAKi treatment, which will guide further studies to establish the optimal usage of JAKi.

## Limitations of the Study

A major limitation of this study was its single-patient design, which was necessitated by the rarity of the disease. To mitigate this limitation, we conducted a longitudinal analysis of the patient's immune landscape and applied multi-omics technologies to comprehensively analyze multiple molecular aspects. While our findings provide valuable insights into the disease pathogenesis, the small sample size may limit the generalizability of our results. There remains a need for further validation studies in larger cohorts to confirm our findings and establish their broader relevance.

### Supplementary Information

Below is the link to the electronic supplementary material.

Supplementary file1 (PPTX 1.70 MB)
