# Supplementary material for: Ruxolitinib Improves Immune-Dysregulation Features but not Epigenetic Abnormality in a Patient with STAT1 GOF
Source: J Clin Immunol. 2024 Apr 5;44(4):84. doi: 10.1007/s10875-024-01687-9 (PMC10997693; doi:10.1007/s10875-024-01687-9)
Supplement: Supplementary file 1 — Supplementary file1 (PPTX 1.70 MB) [file 10875_2024_1687_MOESM1_ESM.pptx]

## Slide 1
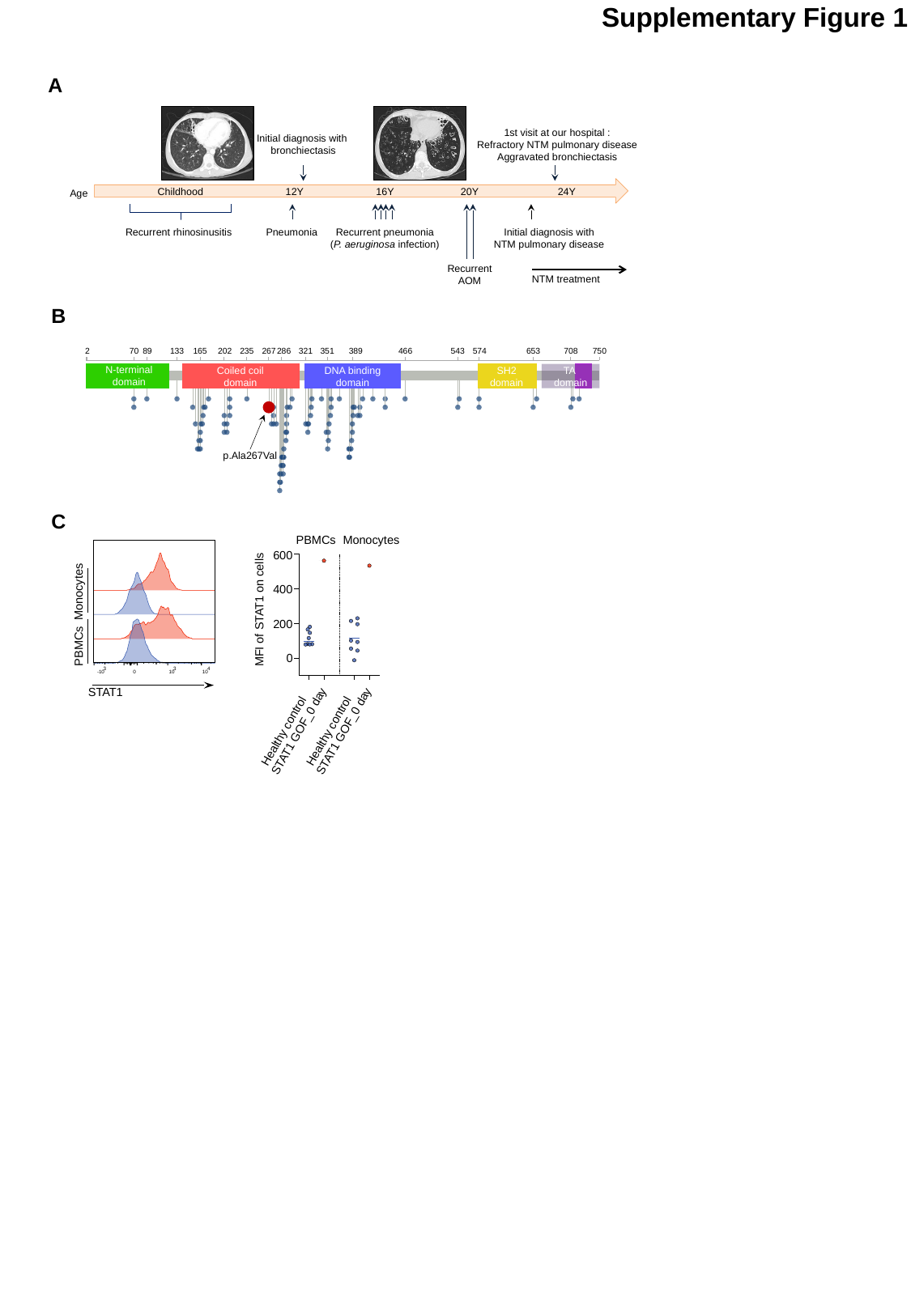

Supplementary Figure 1
A
1st visit at our hospital :
Refractory NTM pulmonary disease
Aggravated bronchiectasis
Initial diagnosis with
bronchiectasis
Age
Childhood
12Y
16Y
20Y
24Y
Recurrent rhinosinusitis
Pneumonia
Recurrent pneumonia
(P. aeruginosa infection)
Initial diagnosis with
NTM pulmonary disease
Recurrent AOM
NTM treatment
B
2
70
89
133
165
202
235
267
286
321
351
389
466
543
574
653
708
750
p.Ala267Val
N-terminal domain
Coiled coil domain
DNA binding domain
SH2
domain
TA
domain
C
PBMCs
Monocytes
600
400
MFI of STAT1 on cells
200
0
Healthy control
STAT1 GOF_0 day
Healthy control
STAT1 GOF_0 day
3
3
4
-10
0
10
10
Monocytes
PBMCs
STAT1

## Slide 2
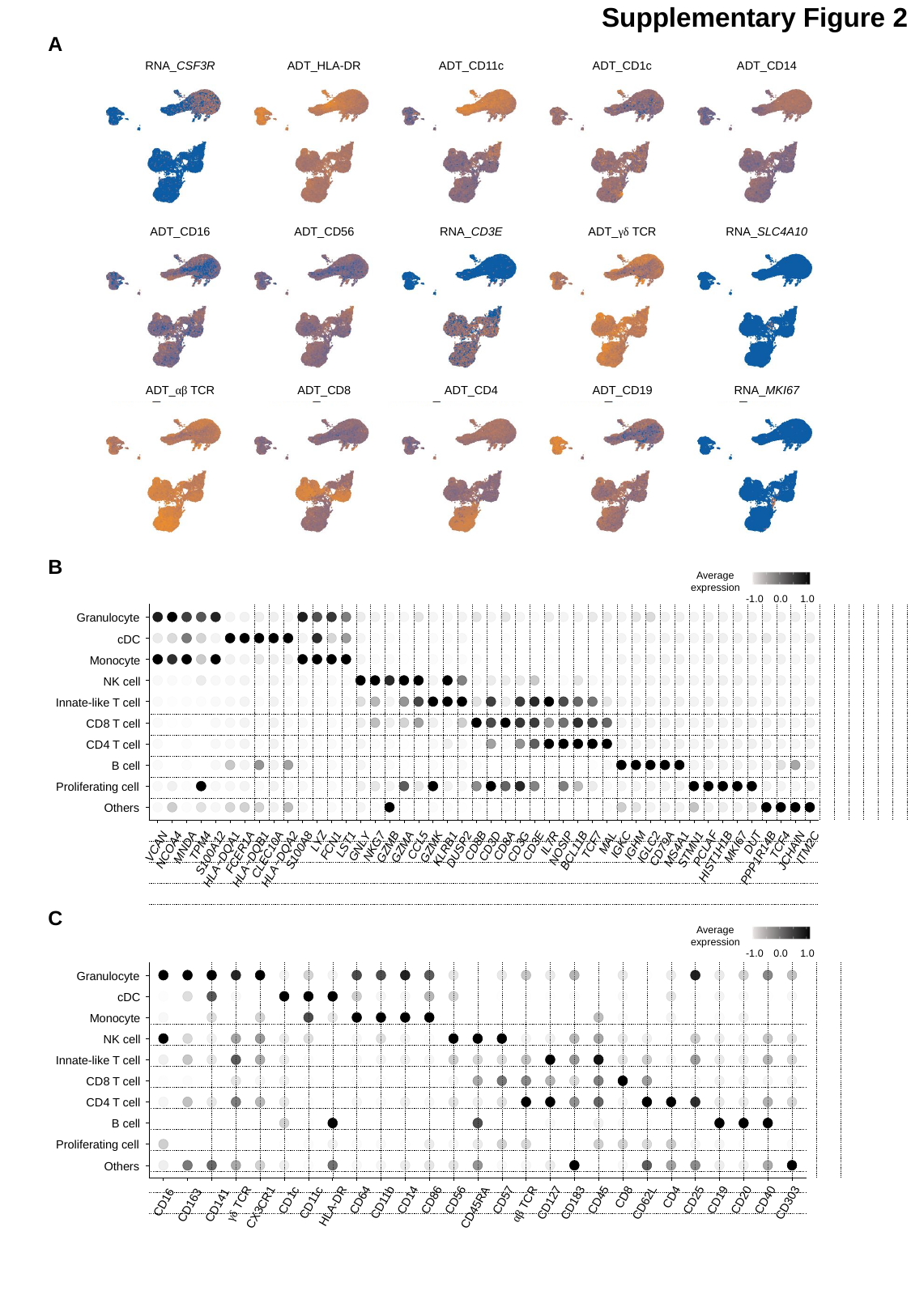

Supplementary Figure 2
A
RNA_CSF3R
ADT_HLA-DR
ADT_CD11c
ADT_CD1c
ADT_CD14
ADT_CD16
ADT_CD56
RNA_CD3E
ADT_γδ TCR
RNA_SLC4A10
ADT_αβ TCR
ADT_CD8
ADT_CD4
ADT_CD19
RNA_MKI67
B
1.0
0.0
-1.0
Average
expression
Granulocyte
cDC
Monocyte
NK cell
Innate-like T cell
CD8 T cell
CD4 T cell
B cell
Proliferating cell
Others
LYZ
FCN1
LST1
TCF7
MAL
IGKC
IGHM
CD79A
MS4A1
STMN1
PCLAF
DUT
TCF4
ITM2C
NCOA4
MNDA
TPM4
S100A12
HLA−DQA1
FCER1A
HLA−DQB1
CLEC10A
HLA−DQA2
S100A8
GZMB
GZMA
CCL5
GZMK
DUSP2
CD8B
CD3D
CD8A
CD3G
CD3E
IL7R
NOSIP
BCL11B
HIST1H1B
PPP1R14B
JCHAIN
GNLY
NKG7
KLRB1
IGLC2
MKI67
VCAN
C
1.0
0.0
-1.0
Average
expression
Granulocyte
cDC
Monocyte
NK cell
Innate-like T cell
CD8 T cell
CD4 T cell
B cell
Proliferating cell
Others
CD16
CD163
CD141
γδ TCR
CX3CR1
CD1c
CD11c
HLA-DR
CD64
CD11b
CD14
CD86
CD56
CD45RA
CD57
αβ TCR
CD127
CD183
CD45
CD8
CD62L
CD4
CD25
CD19
CD20
CD40
CD303

## Slide 3
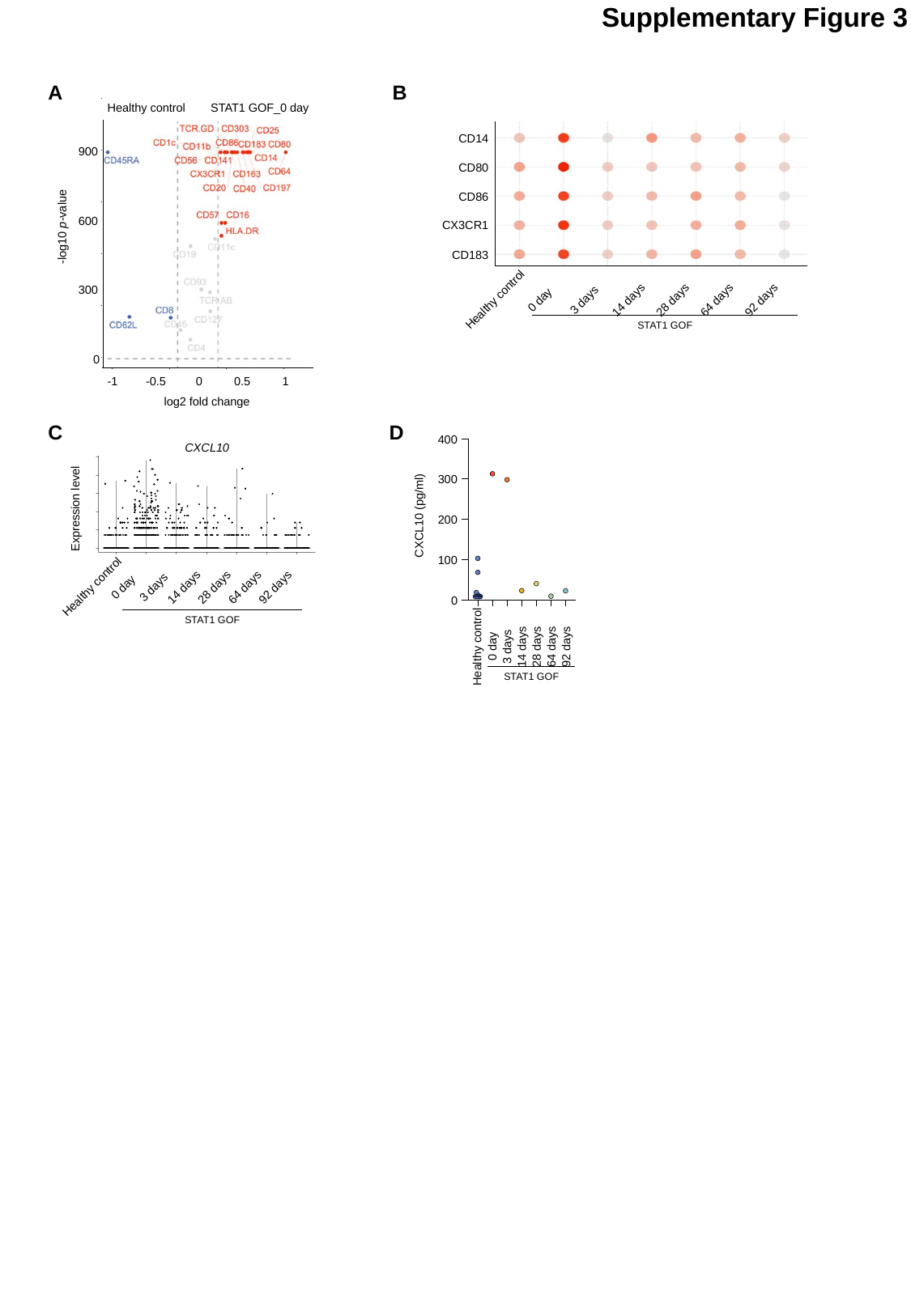

Supplementary Figure 3
A
B
Healthy control
STAT1 GOF_0 day
900
600
300
0
-1
-0.5
0
0.5
1
-log10 p-value
log2 fold change
CD14
CD80
CD86
CX3CR1
CD183
Healthy control
0 day
3 days
14 days
28 days
64 days
92 days
STAT1 GOF
400
300
CXCL10 (pg/ml)
200
100
0
Healthy control
0 day
3 days
14 days
28 days
64 days
92 days
STAT1 GOF
C
D
CXCL10
Expression level
Healthy control
0 day
3 days
14 days
28 days
64 days
92 days
STAT1 GOF

## Slide 4
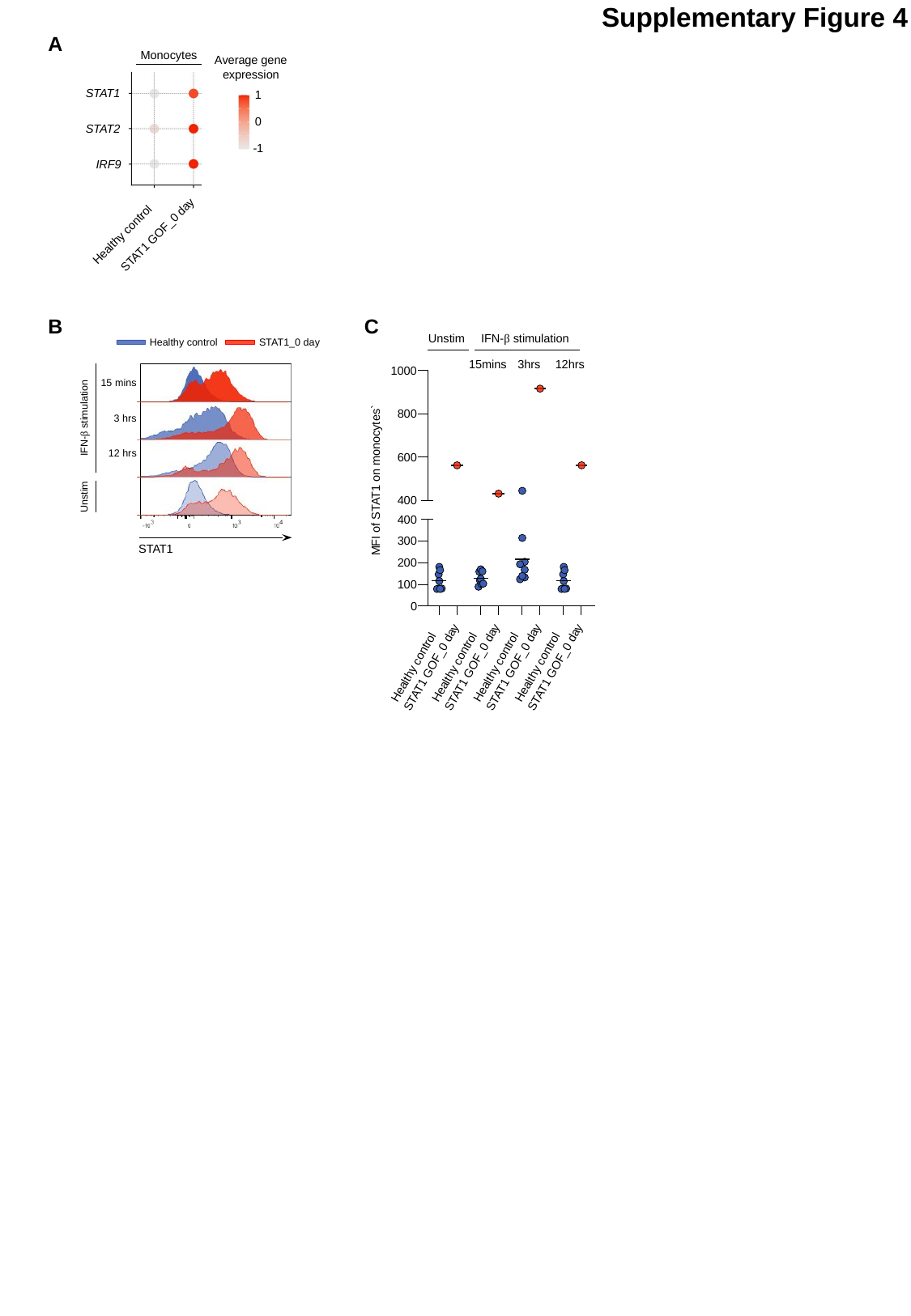

Supplementary Figure 4
A
Average gene
expression
1
0
-1
Monocytes
STAT1
STAT2
IRF9
Healthy control
STAT1 GOF_0 day
B
C
Healthy control
STAT1_0 day
Unstim
IFN-β stimulation
15mins
3hrs
12hrs
1000
800
600
MFI of STAT1 on monocytes`
400
400
300
200
100
0
Healthy control
STAT1 GOF_0 day
Healthy control
STAT1 GOF_0 day
Healthy control
STAT1 GOF_0 day
Healthy control
STAT1 GOF_0 day
15 mins
IFN-β stimulation
3 hrs
12 hrs
Unstim
STAT1
